# Crystal structure of 1,4-bis­(3-ammonio­prop­yl)piperazine-1,4-diium bis­[dichromate(VI)]

**DOI:** 10.1107/S2056989016005284

**Published:** 2016-04-05

**Authors:** S. Vetrivel, E. Vinoth, R. U. Mullai, R. Aruljothi, M. NizamMohideen

**Affiliations:** a PG & Research Department of Physics, Government Arts College, Tiruvannamalai 606 603, Tamil Nadu, India; b Department of Physics, The New College (Autonomous), Chennai 600 014, Tamil Nadu, India

**Keywords:** crystal structure, dichromate anion, piperazinediium cation, mol­ecular salt, hydrogen bonding.

## Abstract

The asymmetric unit of the organic–inorganic title salt, (C_10_H_28_N_4_)[Cr_2_O_7_]_2_, comprises one half of an 1,4-bis­(3-ammonio­prop­yl)piperazinediium cation (the other half being generated by the application of inversion symmetry) and a dichromate anion. The piperazine ring of the cation adopts a chair conformation, and the two CrO_4_ tetra­hedra of the anion are in an almost eclipsed conformation. In the crystal, the cations and anions form a layered arrangement parallel to (001). N—H⋯O hydrogen bonds between the cations and anions and additional C—H⋯O inter­actions lead to the formation of a three-dimensional network structure.

## Abstract 2

The organic-inorganic title salt contains a cation with a chair conformation of the piperazine ring and an eclipsed dichromate anion. The entities are linked by N—H⋯O and C—H⋯O hydrogen bonds into a three-dimensional network structure.

## Chemical context   

Chromium is usually found in trivalent and hexa­valent oxidation states in soil, ground water and seawater (Cespón-Romero *et al.*, 1996 ▸). Trivalent chromium is an essential element in mammals for maintaining efficient glucose, lipid and protein metabolism. On the other hand, hexa­valent chromium is toxic and recognized as a carcinogen to humans and wildlife. Hence the dichromate ion is environmentally important due to its high toxicity (Yusof & Malek, 2009 ▸) and its use in many industrial processes (Goyal *et al.*, 2003 ▸). Recently, the reactions between hexa­ureachromium(III) and inorganic oxoanions (such as Cr_2_O_7_^2−^ or CrO_4_^2−^) in aqueous solution have been investigated (Moon *et al.*, 2015 ▸). Numerous piperazine derivatives have shown a wide spectrum of biological activities, *viz.* anti­bacterial (Foroumadi *et al.*, 2007 ▸), anti­fungal (Upadhayaya *et al.*, 2004 ▸), anti­cancer (Chen *et al.*, 2006 ▸), anti­parasitic (Cunico *et al.*, 2009 ▸), anti­histamine (Smits *et al.*, 2008 ▸) or anti­depressive activities (Becker *et al.*, 2006 ▸). Anti­diabetic, anti-inflammatory, anti­tubercular, anti­malarial, anti­convulsant, anti­pyretic, anti­tumor, anthelmintic and analgesic activities (Gan *et al.*, 2009*a* ▸,*b* ▸; Willems & Ilzerman, 2010 ▸) have also been found to be caused by this versatile moiety. In view of these important properties, we have undertaken the synthesis and X-ray diffraction study of the title compound.
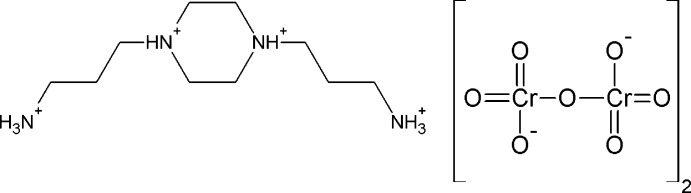


## Structural commentary   

**Figure 1:**
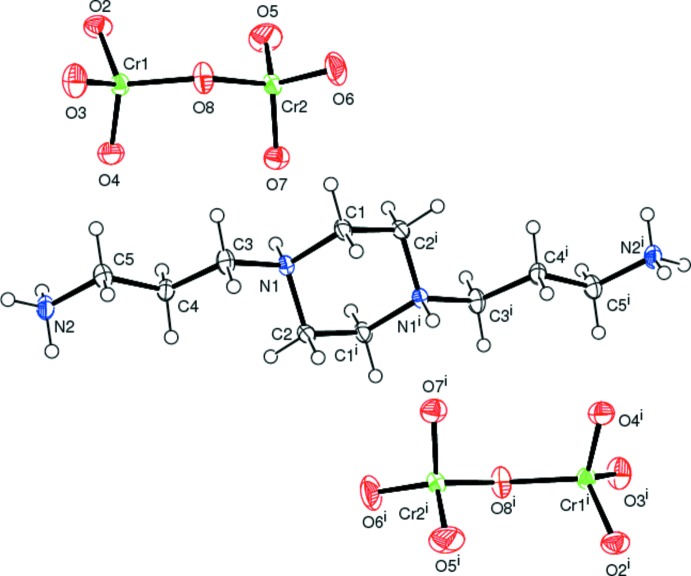
The entities of the organic–inorganic title salt. Displacement ellipsoids are drawn at the 30% probability level. [Symmetry code: (i) −*x* + 2, −*y*, −*z* + 1.]

The mol­ecular entities of the title compound, consisting of a centrosymmetric 1,4-bis­(3-ammonio­prop­yl)piperazinediium cation and a dichromate anion, are shown in Fig. 1 ▸. In the cation, the central piperazine ring (N1/C1/C2/N1^i^/C1^i^/C2^i^; for symmetry operators, see Fig. 1 ▸) is substituted at the two N atoms by two ammonio­propyl moieties. The piperazine ring adopts a chair conformation, as is evident from the puckering parameters: *Q* = 0.599 (2) Å, *τ* = 180.0° and *φ* = 0°. Atoms N1 and N1^i^ are on opposite sides of the C1/C1^i^/C2/C2^i^ plane and are both displaced from it by 0.2446 (19) Å. The chair conformation of the cation in the title structure is very similar to those of the same cation in the crystal structures of the 2-hy­droxy­benzoate (Cukrowski *et al.*, 2012 ▸), the nitrate (Junk & Smith, 2005 ▸) and the tetra­hydrogenpenta­borate (Jiang *et al.*, 2009 ▸) salts, despite the differences in the size and shape of the anions in the various structures. The tetra­hedral CrO_4_ groups in the anion of the title structure are fused together by a common O atom (O8) and are in an almost eclipsed conformation (Brandon & Brown, 1968 ▸). The Cr—O bond lengths follow the characteristic distribution for dichromate anions, with two longer bridging Cr—O bonds of 1.7676 (16) and 1.7746 (15) Å and six shorter terminal Cr—O bonds [range 1.5909 (19)–1.6185 (15) Å]. The Cr1—O8—Cr2 bridging angle in the complex anion is 127.48 (10)°. The tetra­hedral O—Cr—O bond angles [range 106.52 (8) to 112.85 (12)°] indicate slight angular distortions.

## Supra­molecular features   

**Figure 2:**
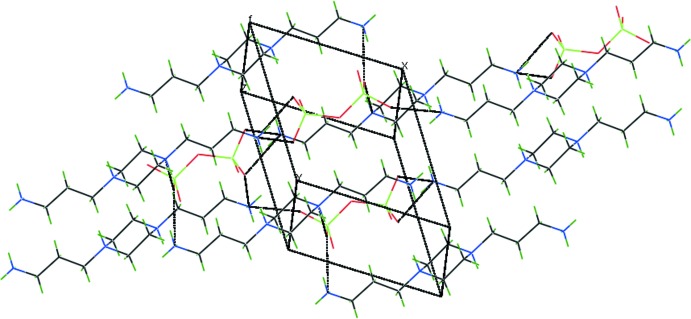
The packing of the mol­ecular entities in the crystal structure of the title salt.

The organic cations and inorganic anions are each arranged in rows parallel to [100] and alternate with each other along [010], forming a layered arrangement parallel to (001). N—H⋯O hydrogen bonds (Table 1 ▸) between the cations, involving both primary and tertiary ammonium groups, and the anions lead to a three-dimensional network structure (Figs. 2 ▸ and 3 ▸). Additional C—H⋯O inter­actions consolidate this arrangement.

## Synthesis and crystallization   

Potassium dichromate and 1,4-bis­(3-amino­prop­yl)piperazine (PDBP) were mixed in a molar ratio of 2:1 in water. Potassium dichromate was first dissolved in Millipore water of 18.2 MΩ·cm resistivity. Then the amount of PDBP was slowly added to the solution together with a few drops of concentrated hydro­chloric acid and the mixture stirred for 18 h. The solution was then filtered twice with Wattmann filter paper and poured into petri dishes to evaporate at room temperature for several days. Recrystallization from water improved the quality of the material and increased the size of the crystals (maximum crystal size 5×3×2 mm^3^ after 35 d). A specimen was cleaved for the present structure determination.

## Refinement   

Crystal data, data collection and structure refinement details are summarized in Table 2 ▸. All hydrogen atoms were placed geometrically and refined using a riding model: N—H = 0.89 Å for the primary ammonium group with *U*_iso_(H) = 1.5*U*_eq_(N); N—H = 0.98 Å for the tertiary ammonium group with *U*_iso_(H) = 1.2*U*_eq_(N); C—H = 0.97 Å with *U*_iso_(H) = 1.2*U*_eq_(C).

## Supplementary Material

Crystal structure: contains datablock(s) global, I. DOI: 10.1107/S2056989016005284/wm5281sup1.cif

Structure factors: contains datablock(s) I. DOI: 10.1107/S2056989016005284/wm5281Isup2.hkl

CCDC reference: 1471068

Additional supporting information:  crystallographic information; 3D view; checkCIF report

## Figures and Tables

**Figure 3:**
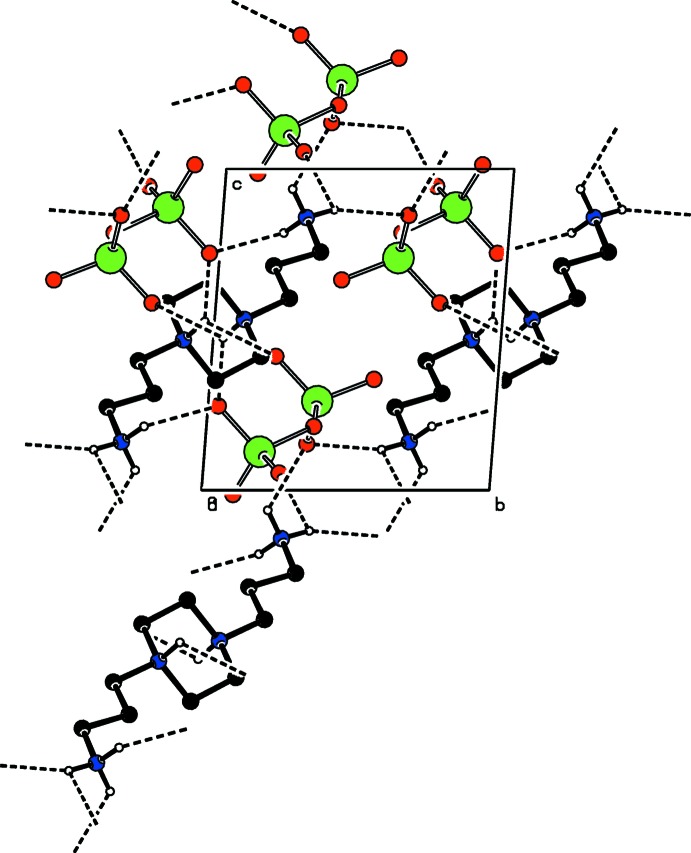
A part of the crystal structure of the title salt in a view along [100] showing N—H⋯O hydrogen-bonding inter­actions as dashed lines. C—H⋯O inter­actions are omitted for clarity.

**Table 1:** Hydrogen-bond geometry (Å, °)

*D*—H⋯*A*	*D*—H	H⋯*A*	*D*⋯*A*	*D*—H⋯*A*
C1—H1*A*⋯O3^i^	0.97	2.28	3.176 (3)	152
C1—H1*A*⋯O4^ii^	0.97	2.61	3.248 (3)	123
C1—H1*B*⋯O6	0.97	2.53	3.353 (3)	143
C2—H2*A*⋯O2^iii^	0.97	2.49	3.298 (3)	141
C2—H2*A*⋯O4^iii^	0.97	2.59	3.061 (3)	110
C2—H2*B*⋯O7^iv^	0.97	2.59	3.232 (2)	124
C3—H3*B*⋯O3^i^	0.97	2.58	3.383 (3)	140
C4—H4*A*⋯O5^v^	0.97	2.38	3.208 (3)	143
C4—H4*B*⋯O7^iii^	0.97	2.64	3.309 (2)	127
N2—H6*A*⋯O2^vi^	0.89	2.18	3.040 (2)	161
N2—H6*B*⋯O7^iii^	0.89	2.05	2.854 (2)	149
N2—H6*C*⋯O2^v^	0.89	2.22	2.865 (2)	129
N2—H6*C*⋯O5^iii^	0.89	2.64	3.239 (3)	125
N1—H1⋯O4^iii^	0.98	2.43	3.113 (2)	126
N1—H1⋯O7	0.98	1.95	2.763 (2)	139

Symmetry codes: (i) 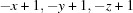; (ii) 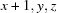; (iii) 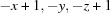; (iv) 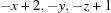; (v) 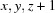; (vi) 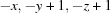.

**Table 2:** Experimental details

Crystal data	
Chemical formula	(C_10_H_28_N_4_)[Cr_2_O_7_]_2_
*M*_r_	636.36
Crystal system, space group	Triclinic, *P* 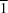
Temperature (K)	293
*a*, *b*, *c* (Å)	8.5361 (3), 8.6272 (3), 8.8576 (3)
α, β, γ (°)	77.761 (1), 72.307 (1), 60.985 (1)
*V* (Å^3^)	541.81 (3)
*Z*	1
Radiation type	Mo *K*α
μ (mm^−1^)	2.03
Crystal size (mm)	0.35 × 0.30 × 0.25

Data collection	
Diffractometer	Bruker Kappa APEXII CCD
Absorption correction	Multi-scan (*SADABS*; Bruker, 2004 ▸)
*T*_min_, *T*_max_	0.528, 0.649
No. of measured, independent and observed [*I* > 2σ(*I*)] reflections	10263, 1913, 1835
*R*_int_	0.020
(sin θ/λ)_max_ (Å^−1^)	0.595

Refinement	
*R*[*F* ^2^ > 2σ(*F* ^2^)], *wR*(*F* ^2^), *S*	0.023, 0.068, 1.06
No. of reflections	1913
No. of parameters	145
H-atom treatment	H-atom parameters constrained
Δρ_max_, Δρ_min_ (e Å^−3^)	0.44, −0.45

Computer programs: *APEX2* and *SAINT* (Bruker, 2004 ▸), *SHELXT* (Sheldrick, 2015*a* ▸), *SHELXL2014/7* (Sheldrick, 2015*b* ▸), *ORTEP-3 for Windows* and *WinGX* (Farrugia, 2012 ▸) and *PLATON* (Spek, 2009 ▸).

## supplementary crystallographic information

### Crystal data

**Table:**

(C_10_H_28_N_4_)[Cr_2_O_7_]_2_	*Z* = 1
*M**_r_* = 636.36	*F*(000) = 324
Triclinic, *P*1	*D*_x_ = 1.950 Mg m^−^^3^
*a* = 8.5361 (3) Å	Mo *K*α radiation, λ = 0.71073 Å
*b* = 8.6272 (3) Å	Cell parameters from 9982 reflections
*c* = 8.8576 (3) Å	θ = 2.4–39.1°
α = 77.761 (1)°	µ = 2.03 mm^−^^1^
β = 72.307 (1)°	*T* = 293 K
γ = 60.985 (1)°	Needle, brown
*V* = 541.81 (3) Å^3^	0.35 × 0.30 × 0.25 mm

### Data collection

**Table:**

Bruker Kappa APEXII CCD diffractometer	1913 independent reflections
Radiation source: fine-focus sealed tube	1835 reflections with *I* > 2σ(*I*)
Graphite monochromator	*R*_int_ = 0.020
ω and φ scan	θ_max_ = 25.0°, θ_min_ = 2.4°
Absorption correction: multi-scan (*SADABS*; Bruker, 2004)	*h* = −10→10
*T*_min_ = 0.528, *T*_max_ = 0.649	*k* = −10→10
10263 measured reflections	*l* = −10→10

### Refinement

**Table:**

Refinement on *F*^2^	0 restraints
Least-squares matrix: full	Hydrogen site location: inferred from neighbouring sites
*R*[*F*^2^ > 2σ(*F*^2^)] = 0.023	H-atom parameters constrained
*wR*(*F*^2^) = 0.068	*w* = 1/[σ^2^(*F*_o_^2^) + (0.0379*P*)^2^ + 0.4739*P*] where *P* = (*F*_o_^2^ + 2*F*_c_^2^)/3
*S* = 1.06	(Δ/σ)_max_ < 0.001
1913 reflections	Δρ_max_ = 0.44 e Å^−^^3^
145 parameters	Δρ_min_ = −0.45 e Å^−^^3^

### Special details

**Table:**

Geometry. All esds (except the esd in the dihedral angle between two l.s. planes) are estimated using the full covariance matrix. The cell esds are taken into account individually in the estimation of esds in distances, angles and torsion angles; correlations between esds in cell parameters are only used when they are defined by crystal symmetry. An approximate (isotropic) treatment of cell esds is used for estimating esds involving l.s. planes.

### Fractional atomic coordinates and isotropic or equivalent isotropic displacement parameters (Å^2^)

**Table:**

	*x*	*y*	*z*	*U*_iso_*/*U*_eq_	
C1	0.9278 (3)	0.1703 (3)	0.4169 (2)	0.0217 (4)	
H1A	0.9548	0.2371	0.4721	0.026*	
H1B	0.8679	0.2498	0.3344	0.026*	
C2	0.8955 (3)	−0.0195 (3)	0.6574 (2)	0.0209 (4)	
H2A	0.8158	−0.0661	0.7319	0.025*	
H2B	0.9217	0.0454	0.7154	0.025*	
C3	0.6197 (3)	0.2538 (3)	0.5974 (3)	0.0247 (4)	
H3A	0.5710	0.3344	0.5101	0.030*	
H3B	0.6391	0.3191	0.6608	0.030*	
C4	0.4805 (3)	0.1911 (3)	0.6986 (2)	0.0218 (4)	
H4A	0.5194	0.1252	0.7946	0.026*	
H4B	0.4723	0.1127	0.6407	0.026*	
C5	0.2943 (3)	0.3494 (3)	0.7415 (3)	0.0259 (4)	
H5A	0.3046	0.4315	0.7931	0.031*	
H5B	0.2522	0.4109	0.6457	0.031*	
N2	0.1592 (2)	0.2910 (2)	0.8492 (2)	0.0261 (4)	
H6A	0.0501	0.3852	0.8736	0.039*	
H6B	0.1488	0.2168	0.8012	0.039*	
H6C	0.1973	0.2358	0.9376	0.039*	
N1	0.8006 (2)	0.1028 (2)	0.53184 (19)	0.0183 (3)	
H1	0.7772	0.0338	0.4742	0.022*	
O2	0.1839 (2)	0.3536 (2)	0.14426 (19)	0.0359 (4)	
O3	0.1272 (2)	0.5696 (2)	0.3451 (2)	0.0402 (4)	
O4	0.2800 (2)	0.2224 (2)	0.41686 (19)	0.0359 (4)	
O5	0.6705 (3)	0.1105 (3)	−0.0133 (2)	0.0612 (6)	
O6	0.8440 (3)	0.2646 (3)	0.0538 (3)	0.0564 (6)	
O7	0.7517 (2)	0.0359 (2)	0.26223 (19)	0.0325 (4)	
O8	0.4835 (2)	0.3730 (2)	0.1987 (2)	0.0370 (4)	
Cr1	0.26337 (4)	0.37840 (4)	0.27693 (4)	0.02227 (12)	
Cr2	0.69282 (5)	0.19141 (5)	0.11987 (4)	0.02748 (13)	

### Atomic displacement parameters (Å^2^)

**Table:**

	*U*^11^	*U*^22^	*U*^33^	*U*^12^	*U*^13^	*U*^23^
C1	0.0192 (10)	0.0226 (9)	0.0222 (10)	−0.0120 (8)	−0.0008 (8)	0.0007 (8)
C2	0.0202 (10)	0.0260 (10)	0.0166 (9)	−0.0124 (8)	−0.0013 (7)	−0.0014 (8)
C3	0.0179 (10)	0.0213 (10)	0.0303 (11)	−0.0073 (8)	0.0011 (8)	−0.0071 (8)
C4	0.0157 (9)	0.0230 (10)	0.0246 (10)	−0.0080 (8)	−0.0029 (8)	−0.0026 (8)
C5	0.0200 (10)	0.0246 (10)	0.0281 (11)	−0.0088 (8)	0.0008 (8)	−0.0051 (8)
N2	0.0163 (8)	0.0313 (9)	0.0275 (9)	−0.0091 (7)	−0.0014 (7)	−0.0056 (7)
N1	0.0155 (8)	0.0201 (8)	0.0190 (8)	−0.0087 (7)	−0.0006 (6)	−0.0042 (6)
O2	0.0460 (10)	0.0430 (9)	0.0291 (8)	−0.0252 (8)	−0.0145 (7)	−0.0021 (7)
O3	0.0322 (9)	0.0346 (9)	0.0514 (10)	−0.0121 (7)	−0.0011 (8)	−0.0190 (8)
O4	0.0387 (9)	0.0418 (9)	0.0313 (8)	−0.0217 (8)	−0.0124 (7)	0.0042 (7)
O5	0.0591 (13)	0.0843 (15)	0.0348 (10)	−0.0178 (12)	−0.0145 (9)	−0.0260 (10)
O6	0.0323 (10)	0.0645 (13)	0.0619 (13)	−0.0286 (9)	0.0011 (9)	0.0150 (10)
O7	0.0384 (9)	0.0356 (9)	0.0321 (8)	−0.0221 (7)	−0.0120 (7)	−0.0006 (7)
O8	0.0250 (8)	0.0364 (9)	0.0484 (10)	−0.0163 (7)	0.0005 (7)	−0.0085 (7)
Cr1	0.02026 (19)	0.0254 (2)	0.02321 (19)	−0.01134 (15)	−0.00346 (13)	−0.00582 (13)
Cr2	0.02130 (19)	0.0382 (2)	0.0210 (2)	−0.01414 (16)	−0.00041 (14)	−0.00334 (15)

### Geometric parameters (Å, º)

**Table:**

C1—N1	1.498 (2)	C5—N2	1.478 (3)
C1—C2^i^	1.502 (3)	C5—H5A	0.9700
C1—H1A	0.9700	C5—H5B	0.9700
C1—H1B	0.9700	N2—H6A	0.8900
C2—N1	1.493 (2)	N2—H6B	0.8900
C2—C1^i^	1.502 (3)	N2—H6C	0.8900
C2—H2A	0.9700	N1—H1	0.9800
C2—H2B	0.9700	O2—Cr1	1.6185 (15)
C3—N1	1.498 (2)	O3—Cr1	1.6035 (16)
C3—C4	1.509 (3)	O4—Cr1	1.6070 (16)
C3—H3A	0.9700	O5—Cr2	1.5909 (19)
C3—H3B	0.9700	O6—Cr2	1.6068 (18)
C4—C5	1.511 (3)	O7—Cr2	1.6299 (16)
C4—H4A	0.9700	O8—Cr2	1.7676 (16)
C4—H4B	0.9700	O8—Cr1	1.7746 (15)
			
N1—C1—C2^i^	111.02 (16)	C4—C5—H5B	109.6
N1—C1—H1A	109.4	H5A—C5—H5B	108.1
C2^i^—C1—H1A	109.4	C5—N2—H6A	109.5
N1—C1—H1B	109.4	C5—N2—H6B	109.5
C2^i^—C1—H1B	109.4	H6A—N2—H6B	109.5
H1A—C1—H1B	108.0	C5—N2—H6C	109.5
N1—C2—C1^i^	110.02 (15)	H6A—N2—H6C	109.5
N1—C2—H2A	109.7	H6B—N2—H6C	109.5
C1^i^—C2—H2A	109.7	C2—N1—C3	113.18 (15)
N1—C2—H2B	109.7	C2—N1—C1	108.55 (15)
C1^i^—C2—H2B	109.7	C3—N1—C1	110.86 (15)
H2A—C2—H2B	108.2	C2—N1—H1	108.0
N1—C3—C4	112.22 (16)	C3—N1—H1	108.0
N1—C3—H3A	109.2	C1—N1—H1	108.0
C4—C3—H3A	109.2	Cr2—O8—Cr1	127.48 (10)
N1—C3—H3B	109.2	O3—Cr1—O4	110.80 (9)
C4—C3—H3B	109.2	O3—Cr1—O2	108.95 (9)
H3A—C3—H3B	107.9	O4—Cr1—O2	109.39 (8)
C3—C4—C5	109.66 (16)	O3—Cr1—O8	106.52 (8)
C3—C4—H4A	109.7	O4—Cr1—O8	108.31 (8)
C5—C4—H4A	109.7	O2—Cr1—O8	112.85 (9)
C3—C4—H4B	109.7	O5—Cr2—O6	112.85 (12)
C5—C4—H4B	109.7	O5—Cr2—O7	107.91 (11)
H4A—C4—H4B	108.2	O6—Cr2—O7	109.68 (9)
N2—C5—C4	110.30 (16)	O5—Cr2—O8	111.08 (10)
N2—C5—H5A	109.6	O6—Cr2—O8	106.39 (10)
C4—C5—H5A	109.6	O7—Cr2—O8	108.89 (8)
N2—C5—H5B	109.6		
			
N1—C3—C4—C5	−171.94 (16)	C2^i^—C1—N1—C3	−176.16 (16)
C3—C4—C5—N2	−176.35 (16)	Cr2—O8—Cr1—O3	175.26 (12)
C1^i^—C2—N1—C3	178.15 (15)	Cr2—O8—Cr1—O4	56.03 (15)
C1^i^—C2—N1—C1	−58.3 (2)	Cr2—O8—Cr1—O2	−65.22 (15)
C4—C3—N1—C2	−64.5 (2)	Cr1—O8—Cr2—O5	52.36 (17)
C4—C3—N1—C1	173.31 (16)	Cr1—O8—Cr2—O6	175.53 (13)
C2^i^—C1—N1—C2	58.9 (2)	Cr1—O8—Cr2—O7	−66.33 (14)

Symmetry code: (i) −*x*+2, −*y*, −*z*+1.

### Hydrogen-bond geometry (Å, º)

**Table:**

*D*—H···*A*	*D*—H	H···*A*	*D*···*A*	*D*—H···*A*
C1—H1*A*···O3^ii^	0.97	2.28	3.176 (3)	152
C1—H1*A*···O4^iii^	0.97	2.61	3.248 (3)	123
C1—H1*B*···O6	0.97	2.53	3.353 (3)	143
C2—H2*A*···O2^iv^	0.97	2.49	3.298 (3)	141
C2—H2*A*···O4^iv^	0.97	2.59	3.061 (3)	110
C2—H2*B*···O7^i^	0.97	2.59	3.232 (2)	124
C3—H3*B*···O3^ii^	0.97	2.58	3.383 (3)	140
C4—H4*A*···O5^v^	0.97	2.38	3.208 (3)	143
C4—H4*B*···O7^iv^	0.97	2.64	3.309 (2)	127
N2—H6*A*···O2^vi^	0.89	2.18	3.040 (2)	161
N2—H6*B*···O7^iv^	0.89	2.05	2.854 (2)	149
N2—H6*C*···O2^v^	0.89	2.22	2.865 (2)	129
N2—H6*C*···O5^iv^	0.89	2.64	3.239 (3)	125
N1—H1···O4^iv^	0.98	2.43	3.113 (2)	126
N1—H1···O7	0.98	1.95	2.763 (2)	139

Symmetry codes: (i) −*x*+2, −*y*, −*z*+1; (ii) −*x*+1, −*y*+1, −*z*+1; (iii) *x*+1, *y*, *z*; (iv) −*x*+1, −*y*, −*z*+1; (v) *x*, *y*, *z*+1; (vi) −*x*, −*y*+1, −*z*+1.
